# Exit Gluten-Free and Enter Low FODMAPs: A Novel Dietary Strategy to Reduce Gastrointestinal Symptoms in Athletes

**DOI:** 10.1007/s40279-018-01034-0

**Published:** 2019-01-22

**Authors:** Dana M. Lis

**Affiliations:** Department of Neurobiology, Physiology and Behavior, University California Davis, Davis, CA 95616 USA

## Abstract

Exercise-associated physiological disturbances alter gastrointestinal function and integrity. These alterations may increase susceptibility to dietary triggers, namely gluten and a family of short-chain carbohydrates known as FODMAPs (fermentable oligo-, di-, monosaccharides and polyols). A recent surge in the popularity of gluten-free diets (GFDs) among athletes without celiac disease has been exacerbated by unsubstantiated commercial health claims and high-profile athletes citing this diet to be the secret to their success. Up to 41% of athletes at least partially adhere to a GFD diet, with the belief that gluten avoidance improves exercise performance and parameters influencing performance, particularly gastrointestinal symptoms (GIS). In contrast to these beliefs, seminal work investigating the effects of a GFD in athletes without celiac disease has demonstrated no beneficial effect of a GFD versus a gluten-containing diet on performance, gastrointestinal health, inflammation, or perceptual wellbeing. Interestingly, the subsequent reduction in FODMAPs concurrent with the elimination of gluten-containing grains may actually be the factors affecting GIS improvement, not gluten. Pre-existent in the gastrointestinal tract or ingested during exercise, the osmotic and gas-producing effects of variably absorbed FODMAPs may trigger or increase the magnitude of exercise-associated GIS. Research using FODMAP reduction to address gastrointestinal issues in clinically healthy athletes is emerging as a promising strategy to reduce exercise-associated GIS. Applied research and practitioners merging clinical and sports nutrition methods will be essential for the effective use of a low FODMAP approach to tackle the multifactorial nature of gastrointestinal disturbances in athletes.

## Key Points

**Table:** 

For athletes not diagnosed with a clinical condition requiring a gluten-free diet (GFD), this diet does not impart a beneficial or negative effect on performance, gastrointestinal health, or wellbeing. Factors such as nutritional adequacy and subsequent dietary changes should be evaluated when a GFD is considered.
Some fermentable oligo-, di-, monosaccharides and polyols (FODMAPs) may co-exist in gluten-containing foods. A reduction in FODMAPs, rather than gluten, may be the factors influencing gastrointestinal symptom (GIS) improvement. Athletes following a GFD likely inadvertently reduce high FODMAP foods, which may reduce GIS.
Guided by a qualified sports nutrition practitioner, FODMAP restriction may be an effective strategy to reduce the magnitude of exercise-associated GIS.

## Gastrointestinal Symptoms (GIS) in Exercise

Exercise-induced gastrointestinal syndrome, a recently coined term, describes disturbances of gastrointestinal integrity and function that are common features of strenuous exercise [1]. Gastrointestinal conditions are recognized as the most commonly reported illnesses at international sporting events [2]. Prevalence rates range from 30 to 50% of athletes, and up to 90% in ultra-endurance events [3]. Moderate to severe gastrointestinal symptoms (GIS) of the upper and lower gastrointestinal tract may be detrimental to athletic performance, most notably during strenuous endurance exercise. GIS triggered by the physiological responses to exercise are instigated through two main pathways: (1) a circulatory-gastrointestinal pathway where blood flow is redistributed away from the splanchnic area to peripheral circulation and contracting muscles (termed splanchnic hypoperfusion); and (2) a neuroendocrine-gastrointestinal pathway characterized by increased sympathetic nervous system activation [4, 5]. Intestinal ischemia, resulting from splanchnic hypoperfusion, disrupts the epithelial barrier and increases intestinal permeability, allowing an upsurge of bacterial translocation and local and systemic inflammatory responses [6, 7]. Alterations in gut motility and oral rectal transit may also result from this exercise-triggered sympathetic response [8, 9]. Additionally, exercise-associated upper and lower GIS are influenced by foods and fluids consumed around exercise [10, 11].

### Dietary Strategies to Reduce GIS

Various nutrition strategies have been implemented with the aim to reduce exercise-associated GIS in training and competition [11–14]. Pre-exercise diet recommendations to attenuate symptoms during exercise include reduced fiber/low residue, low fat, moderate protein, and avoidance of lactose-containing dairy products. The transient nature and difficulty in reproducing GIS present a challenge in determining the efficacy of these strategies. Consequently, athletes pursue a variety of dietary approaches aimed at reducing GIS. Anecdotal-based approaches or implementation of strategies ad hoc abound, but several evidence-based strategies for reducing GIS have been established [11–14]. One example is ingesting multiple transporter carbohydrates (e.g. glucose and fructose blends) during exercise [15]. More recently, an investigation in runners (six males, five females) demonstrated that carbohydrate and protein intake during exercise under conditions of heat stress (2 h run at 60% maximal oxygen uptake in a 35 °C environment) ameliorated intestinal injury and permeability and decreased GIS with the carbohydrate feeding intervention [16]. Gut training to increase carbohydrate tolerance and prepare race-day nutrition has also been shown to be beneficial [11, 17, 18]. Mechanisms potentiating nutrient malabsorption, such as intestinal injury or decreased intestinal transporter activity, require further consideration when implementing strategies to address exercise-induced gastrointestinal syndrome [19]. Symptoms often persist despite implementation of a variety of strategies.

Being the body’s largest immune organ, the gut is central to immune function, which is further influenced by dietary intake and exercise [20, 21]. It is plausible that stress placed on the gut occurring more frequently than the 3–5 days required for epithelial cell protein turnover [22] generates a state with various levels of perpetual intestinal injury. This circumstance may increase susceptibility to dietary triggers (e.g. gluten) or food intolerances (e.g. lactose), associated GIS, and the long-term development of chronic disease [1]. Although this concept has not been directly demonstrated in athletes, better understanding of dietary factors affecting GIS [e.g. fermentable oligo-, di-, monosaccharides and polyols (FODMAPs)] has improved the practitioner’s toolbox for symptom management [23]. Bridging clinical and sports nutrition will become increasingly fundamental for the efficacious management of exercise-induced gastrointestinal syndrome and gastrointestinal conditions in athletes.

### Objectives

There are two main aims for this review. The first aim is to provide the sports nutrition practitioner with an overview of the current state of knowledge and considerations pertaining to the appropriateness of a gluten-free diet (GFD) for athletes without a clinical requirement for this diet. The second aim is to review the use of FODMAP restriction in clinically healthy athletes to decrease GIS.

## Gluten-Free Diet (GFD) Considerations

### Gastrointestinal Health

A high number of athletes link exercise-associated GIS with dietary triggers, particularly gluten-containing foods [14]. As a result, foods or food groups are eliminated from the diet, potentially unnecessarily. Gluten-containing foods are commonly blamed for GIS. Gluten, a plant storage protein, is a composite of gliadins and glutenins, complex proteins unusually rich in prolines and glutamines that are incompletely digested by intestinal enzymes [24]. It is also important to acknowledge that gluten and fructans co-exist in cereals and yet gluten has been incorrectly blamed for related GIS [25–27]. In celiac disease, partial peptide digestion triggers increased intestinal permeability and innate and/or adaptive immune responses resembling exposure to gastrointestinal pathogens [28]. Non-celiac gluten/wheat sensitivity (NCGS) is acknowledged as a separate clinical entity, with symptoms similar to celiac disease. NCGS is characterized by variable pathogenesis, clinical history, and clinical course [29]. In other words, it is a heterogeneous condition but has symptom overlap with other conditions, such as irritable bowel syndrome (IBS). Advancing work is narrowing the clinical picture for NCGS and suggests a central role of the intestinal innate immune system and elevated levels of intestinal fatty acid binding protein [30, 31]. However, due to the lack of a diagnostic biomarker for NCGS, non-specific symptoms, as well as the resemblance to gastrointestinal and extraintestinal symptoms associated with strenuous exercise (e.g. fatigue, bloating, diarrhea), many athletes believe gluten to be the cause.

A recent questionnaire-based study quantified athletes’ (*n* = 910) beliefs and reasons for adhering to a GFD [14]. Forty-one percent of the non-celiac athletes surveyed reported following this diet [14, 32] at least 50% of the time [14]. The foremost rationale for athletes to adhere to a GFD was self-diagnosis of gluten-related conditions or non-clinical motivations (e.g. healthier, improved body composition) [14]. Observationally, athletes often implement dietary strategies ahead of supportive research, which may or may not be accurate. It is however prudent to consider a potential interplay between exercise-induced gastrointestinal injury, gastrointestinal dysfunction, and susceptibility to the negative effects of known dietary triggers, in this case gluten.

Average intakes of gluten vary individually and geographically [33]. Athletes’ consumption of gluten-containing foods may be higher than average due to the increased volume of food and possibly higher reliance on wheat-based foodstuff (e.g. energy bars, breads, and pasta) to fuel elevated energy and carbohydrate demands. The combination of increased exercise-induced gastrointestinal permeability and high intakes of gluten peptides could allow for a greater gliadin peptide translocation across the epithelial barrier. This permutation of physiological stress and consumption of a known dietary trigger could amplify GIS associated with exercise-induced gastrointestinal syndrome. In vivo, this concept is speculative, and current research is limited to cell culture studies or populations with celiac disease that have demonstrated that gliadin peptides trigger, or are associated with, tight junction protein dysregulation [34, 35]. In the only randomized controlled trial to investigate athletes’ (*n* = 13) gastrointestinal injury and symptoms in response to a GFD during strenuous exercise, gluten did not increase exercise-induced alterations in epithelial injury or GIS [36]. While the alleged detrimental effects of gluten on gut barrier function or GIS in healthy athletes have not been validated, a multitude of physiological elements and psychosocial factors influence GIS and the perception of gluten’s effect on gastrointestinal health.

### Inflammation

In addition to exercise stress, the potential of gluten to trigger inflammatory responses could have an additive toll on the immune system [1]. Immune-associated symptoms are difficult to isolate, yet the influential role of nutrition on immune parameters is an integral component of athletic health and performance. A ‘J-shaped curve’ model has been used to demonstrate the relationship between high-volume/intensity exercise and increased illness rate [37]. In celiac disease, a GFD restores innate and adaptive immune parameters [38]. However, no evidence supports the idea that gluten avoidance in clinically healthy athletes improves measures of immune function [36, 39]. In a 7-day randomized crossover GFD intervention study of competitive non-celiac cyclists (*n* = 13), gluten did not elicit an adverse inflammatory profile (interleukin (IL)-1β, IL-6, IL-8, IL-10, IL-15, tumor necrosis factor-α) pre, during, or after a strenuous cycling bout (45-min steady-state cycling at 80% maximum power at VO_2max_, followed by a 15-min time trial) compared with an isocaloric gluten-containing diet [36]. Nutrition strategies that improve immune function are desirable, particularly during heavy training periods. However, too little is known about the effect of gluten on immune health to support adherence to a GFD for clinically healthy athletes. Future work should continue to evaluate the unique connection between a GFD and the body’s cornerstone for immune health, the gut [14].

### Confounding Dietary Changes

Perceptions of improved perceptual wellbeing [14] are likely influenced by dietary changes concurrent with following a special diet, and not gluten itself. Following a GFD may increase awareness of food choices and encourage more fruit, vegetable, and gluten-free whole-grain intake [14]. These are healthy eating guidelines that align with foundational sports nutrition recommendations [40]. Positive dietary changes associated with this diet are likely to influence perceptions of improved health, psychology, or exercise performance. The ‘belief effect’ is also expected to influence performance outcomes [41] as many athletes trust that a GFD provides an ergogenic edge [14, 32] and has a net positive impact on performance outcomes [41]. However, when 13 trained cyclists were blinded, no difference in a 15-min time-trial performance was found between a gluten-containing diet (245.4 ± 53.4 kJ) and a GFD (245.0 ± 54.6 kJ) [36]. Widespread convictions of the overall health and performance benefits of a GFD are not well-supported in athletes not requiring gluten avoidance. In a few cases, the ‘belief effect’ as it relates to a GFD may be a strategic ergogenic tool; however, these circumstances should be carefully assessed.

### Effects on Body Composition

In many endurance and aesthetic sports, body composition is an important factor in performance outcomes. Weight changes before and after adherence to a GFD have been examined in celiac disease. However, poor dietary control, methodological differences, and a complexity of confounding factors (e.g. type 1 diabetes) limit the applicability of findings to healthy athletes without celiac disease [42, 43]. A possible increased risk of obesity with adherence to a GFD in celiac disease populations is suggested to be linked to improved nutrient absorption upon villus recovery. Intakes of gluten-free specialty products, historically higher in fat and sugar than their gluten-continuing counterparts, may also contribute to increased obesity [43]. Research in male mice suggests some metabolic differences pertaining to adiposity and metabolism with a GFD versus an isocaloric gluten-containing diet over 8 weeks [44, 45]. Impaired glucose homeostasis, decreased fasted and non-fasted oxygen uptake, lowered energy expenditure, and increased adipocyte content and proinflammatory cytokines were associated with increased body weight and adipose tissue in gluten-fed mice [45]. Upregulated expression of some genes (e.g. peroxisome proliferator-activated receptor-α and -γ, lipoprotein lipase) and hormone concentrations (e.g. leptin, resistin) provide some mechanistic insight [45]. Although these in vitro findings provide an interesting rationale, it would be far-reaching to accept that gluten would elicit the same alterations in clinically healthy athletes. Dietary changes and confounding factors associated with a GFD may be more potent influencers of energy balance and body composition.

### Nutritional Adequacy of a GFD for Athletes

Only approximately 1% of Americans have diagnosed celiac disease [46]. According to the National Health and Nutrition Examination Survey (2009–2014; *n* = 7417), 25% of American consumers reported consuming gluten-free foods in 2015, representing a 67% increase from 2013 [46, 47]. GFD adherence in athletes is estimated to be fourfold higher than the proportion of the general population that is considered to require gluten-avoidance for clinical reasons (e.g. celiac disease, wheat allergy, NCGS) [14]. As mentioned, positive dietary behaviors may accompany a GFD. However, unnecessary food restriction is concerning for athletes given increased fuel requirements and the importance of adequate nutrient intake on health, training adaptation, and performance [14, 40, 48]. Elimination-type diets pose a risk of suboptimal fueling and uncertainty remains whether a GFD is a healthier or less healthy diet to support athlete nutrition demands [49]. Previous studies have found no difference in energy intake with a GFD compared with control diets. However, suboptimal intake of fiber, vitamin D, vitamin B12, folate, iron, zinc, magnesium, and calcium have been reported [42, 50]. With one-quarter of Americans eating gluten-free foods, it is also speculated that consuming alternative rice-based grains may increase exposure to higher levels of arsenic and mercury [47]. In many parts of the world, a proliferating gluten-free market has improved the availability of more nutrient-rich pseudo-cereal-based products, such as amaranth, buckwheat, and quinoa. These are replacing less nutritious corn and rice flour and reducing suboptimal nutrient concerns [51]. However, athletes with modest nutrition knowledge, limited capacity to finance more expensive gluten-free food products [48], or travelling abroad for training/competition may still face food accessibility challenges. Alongside unique athletic fueling requirements, the socioeconomical, psychological, and logistical implications associated with a GFD should be considered when determining the appropriateness of the worlds most popular diet for athletes [52, 53]. An individualized and evidence-based approach to a GFD is advised to optimize nutrition intake to support optimal wellbeing, training adaptation, and performance.

**Fig. 1:**
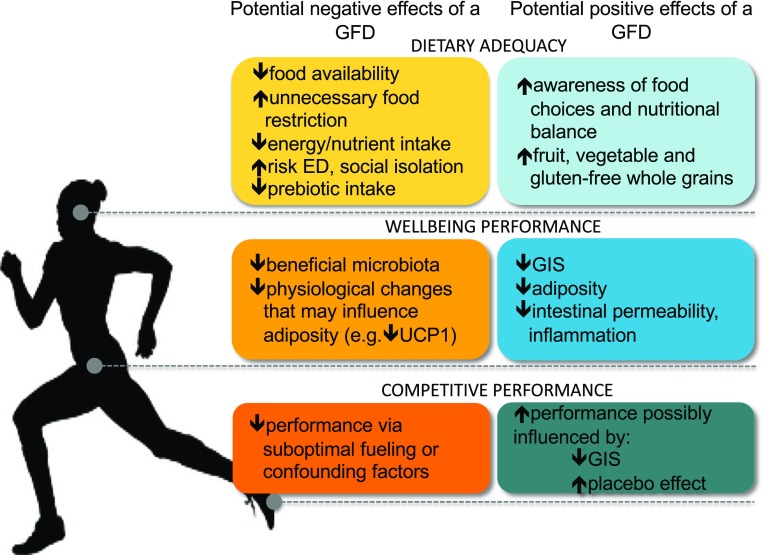
Schematic overview of the potential negative or positive effects/interactions of a GFD as it pertains to athlete performance or health. *ED* eating disorder, *GFD* gluten-free diet, *UCP1* uncoupling protein 1, *GIS* gastrointestinal symptoms. Modified from Lis et al. [73], with permission

The appropriateness of a GFD for clinically healthy athletes should consider several factors (Fig. 1). One of the most important features is that some athletes use avoidance of gluten-containing foods to conceal restrained eating behaviors and eating disorders, particularly in weight-dependent sports [49, 54]. Orthorexic behaviors may also become more complicated with the belief that a GFD is healthier [14]. Eating gluten-free may become such a focal point that the importance of consuming a balanced diet supportive of training and recovery is overlooked. Complications possibly arising from adherence to a restrictive diet while aiming to balance the demands of athletic training include increased time commitment involved in shopping, preparing gluten-free meals, and expense. Additional food-related anxiety, social concerns, and interference with appropriate nutritional/medical guidance are also concerns related to this diet [48, 55, 56]. An awareness of the known associations of elimination diets with restrictive eating behaviors and evaluation of all potential benefits and risks of going gluten-free is prudent (Fig. 1) [49, 54, 55].

### Link Between a GFD and FODMAPs

An emerging trend of athletes self-diagnosing NCGS has been partially underpinned by the lack of a definitive diagnostic biomarker [52, 57, 58]. As mentioned, advancing work proposes that a unique systemic immune response to microbial and wheat antigens, together with intestinal cell damage, occurs in NCGS [59]. Until validation of established biomarkers becomes possible, athletes may continue to attribute gastrointestinal issues to gluten. However, other proteins or nutrients existing in wheat-based foods could be the actual culprits. A previous review has extensively summarized the potential for other proteins existing in gluten-containing grains, mainly cereal protein amylase-trypsin inhibitors, to trigger GIS [60]. Amassing evidence indicating that FODMAPs play a role in augmenting exercise-induced GIS suggests that this frontline dietary management for IBS may cross over to address GIS in athletes [1, 13, 61, 62]. Certain FODMAPS are subsequently reduced with the reduction of wheat-based grains. A decrease in FODMAP intake and not gluten itself may be the true factor for improved GIS attributed to a GFD [63]. This has been well-demonstrated in a handful of clinical studies indicating that fructan and not gluten elimination reduced GIS in IBS patients with self-reported NCGS [26, 63, 64]. This promising area of work may open an entirely novel dietary strategy to manage an extremely common illness among athletes.

## GIS Crossover Between Irritable Bowel and Exercise-Induced Gastrointestinal Syndrome

As a first-line treatment for IBS, a low FODMAP diet has become a widely established strategy for efficacious reduction of GIS [65–69]. These carbohydrates are consequently cosmetically active and fermented in parts of the GI tract [70]. Variable digestion occurs due to either the incomplete absorption (e.g. monosaccharides such as fructose, or polyols such as sorbitol) or the lack/reduced concentration of a specific hydrolase enzyme (e.g. fructans, galacto-oligosaccharides (GOS); see Staudacher et al. for a review of digestion of FODMAPs and the low FODMAP diet for IBS [70]). Variable digestion of these carbohydrates in the small intestine, and fermentation in the colon, potentially elicits adverse GIS in IBS. Interestingly, symptoms related to IBS are analogous to those reported in exercise-induced gastrointestinal syndrome, with diverse mechanisms [10]. Collective symptoms include (1) upper GIS with upper abdominal bloating, regurgitation, belching, nausea, epigastric pain, and heartburn; and (2) lower GIS with lower abdominal bloating, abdominal pain, abnormal, flatulence, urge to defecate, diarrhea and/or defecation, including loose watery stools and fecal blood loss [1, 71].

### FODMAP Elimination

A recent questionnaire-based study reported that 55% of athletes (*n *= 910) self-report eliminating at least one food high in FODMAPs with the aim to reduce GIS [13]. Subsequently, up to 85% reported symptom improvement with removal of the offending food. Lactose (86.5%) was most frequently eliminated, followed by GOS (23.9%), fructose (23.0%), fructans (6.2%), and polyols (5.4%). A case study report of a clinically healthy competitive multisport athlete with persistent exercise-induced GIS similarly confirmed FODMAP restriction compared with a high FODMAP diet (7.2 ± 5.7 g vs. 81.0 ± 5.0 g·FODMAP·day^−1^) measurably reduced daily and during exercise GIS. Lactose intake was presumably the most ubiquitous symptom contributor [61]. Correspondingly, athletes with IBS, estimated to account for 22% of endurance athletes [72], would likely benefit from FODMAP restriction, both for daily and sport-specific fueling.

### Rationale for a Low FODMAP Diet Approach

The efficacy of FODMAP modification for the treatment of exercise-induced GIS in clinically healthy endurance athletes is emerging as a novel approach to attenuate symptoms [1, 73]. The physiological alterations occurring with exercise-induced gastrointestinal syndrome may impair nutrient absorption, alter gastric/intestinal motility, and impair overall gastrointestinal function. Impaired digestion and absorption of FODMAPs may contribute to symptoms related to this multifactorial condition. Adverse lower GIS appear to be dominant in exercise and may be associated with the osmotic effect of these malabsorbed carbohydrates, which can increase luminal water content first in the small intestine [74]. Undigested FODMAPs then travel to the ileum and onwards, where rapid bacterial fermentation may increase intestinal luminal pressure by increasing colonic content through gas production (e.g. H_2_, CH_4_, CO_2_, and H_2_S) and osmotic water translocation. Subsequently, lower GIS are triggered or amplified [75, 76]. As a result, bloating, abdominal pain, flatulence and alterations in bowel movement may occur with greater severity in hypersensitive individuals [70, 75]. FODMAPs also influence upper GIS, such as the sensation of fullness, as demonstrated in a clinical feeding study that administered doses of fructose and glucose via gastric infusion [77]. FODMAPs are not likely to be an exclusive GIS trigger but may amplify symptoms initiated by other factors [1, 62].

### A Modified Low FODMAP Strategy for Exercise-Associated GIS

IBS-like symptoms experienced by many athletes are likely induced by the mechanical, psychological, environmental, and nutritional components related to strenuous exercise. Several dietary strategies may decrease the magnitude of these symptoms. Recently, a short-term low FODMAP diet has been shown to significantly reduce daily GIS in clinically healthy runners with exercise-associated GIS [62]. This randomized, blinded, crossover study assessed the efficacy of a short-term low FODMAP diet (6 days in total), including 2 days of prescribed high-intensity running, on daily and during exercise GIS compared with a high FODMAP diet. A significantly smaller area under the curve for daily GIS was demonstrated in 80% of subjects with consumption of a low compared with a high FODMAP diet [62]. During exercise in the two conditions, symptoms were not significantly different and it is probable that an exercise bout of longer duration or greater intensity may be required to detect measurable differences in GIS during exercise between the two diets [e.g. 2 h running at 60% maximal oxygen uptake (*V*O_2max_)]. Results from this study verify previous case study outcomes and establish the foundation for future research [61]. Forthcoming investigations of the efficacy of FODMAP modification in athletic populations should aim to determine the ideal timing and amount of FODMAP intake around strenuous exercise while maintaining a focus on minimizing the risks associated with unnecessary food restriction.

### High FODMAP Food Ingestion During Exercise

A limited number and variety of specialty sport foods (e.g. energy bars, gels) have been analyzed for FODMAP content. All of the products analyzed to date are classified as high FODMAP (Low FODMAP Diet App, Monash University, Melbourne, VIC, Australia). FODMAPs that are common ingredients in sports foods include dried dates, oats, almonds, fructose, mannitol, inulin, apple juice concentrate, chicory root, honey, and others (Table 1). Especially when sports foods are consumed in accordance with sports nutrition guidelines, high amounts of FODMAPs are liable to be ingested [40, 61, 62], and potentially malabsorbed, with the resultant amplification of GIS. For example, osmotic effects may contribute to loose, watery stools. Increased colonic gas volume may amplify symptoms such as lower abdominal bloating and pain. Gastrointestinal dysfunction and malabsorption of FODMAPs may increase breath hydrogen and methane excretion. Breath testing is an assessment tool to measure carbohydrate malabsorption, and is used to explore the pathophysiology of functional gastrointestinal disorders [78, 79].

**Table 1:** High FODMAP foods and low FODMAP alternatives commonly consumed in an athlete’s diet

FODMAP categories	High FODMAP foods^a^	Low FODMAP food exchanges^b^
High lactose	Yogurt, cow’s milk	Lactose-free milk, soy milk (from soy protein)
Excess fructose	Apples, figs, watermelon, cherries, agave, honey, many fruit juices (e.g. apple), beetroot juice with apple juice included/whole beetroot	Oranges, berries, bananas, grapes, kiwifruit, cantaloupe, strawberries, blueberries, raspberries, blended vegetable juice (tomato-based) canned or pickled beets
High fructans/galacto-oligosaccharides	Dates, cashews/pistachio nuts, breads/bagels, onions, wheat-based energy bars	Gluten-free, spelt, special sourdough spelt breads, rice cakes, corn tortillas, wheat and/or gluten-free energy bars
High polyols	Dried apricots, protein bars and powders, some electrolyte tablets, sugar-free gum/candies	Protein bars with alternative sweeteners, limit intake of sugar-free gum/candies or choose sugar-containing brands

*FODMAP* fermentable oligo-, di-, monosaccharides and polyols

^a^Check cereals, bars, sports foods, and mixed meals for high FODMAP ingredients

^b^Low FODMAP diets should be guided by a sports dietitian. Sports dietitians advising on low FODMAP diets should be guided by the Monash Low FODMAP Diet course^®^ [74, 88]

A small number of studies have used breath testing to measure carbohydrate malabsorption during and post exercise, with variable results [11, 79, 80]. During mixed endurance exercise (3 h at 75% *V*O_2max_), breath hydrogen was higher during running than during cycling, with ingestion of glucose-rich carbohydrates in semi-solid and fluid forms (approximately 1.2 g·kg body mass·h^−1^), resulting in negligibly higher breath hydrogen excretion (2–3 ppm increase) compared with a non-carbohydrate placebo [79]. It is likely that increased ventilation rates accompanying exercise may skew gas (hydrogen) production values, and the ecological significance is unspecified. Measures taken during the recovery time course after exercise may be more accurate. In a gut adaptability study, after 3 h of running (2 h at 60% *V*O_2max_, followed by a 1 h distance test), 68% of runners demonstrated evidence of carbohydrate malabsorption (breath hydrogen ≥ 10 ppm above baseline) during the recovery period [11]. Forthcoming research will better characterize FODMAP malabsorption during and after exercise to better inform food selection. However, hydrogen breath testing has demonstrated poor reproducibility and low predictive value for symptom responses to lactose and fructose [78]. Variable absorption of FODMAPs should be considered a potential factor contributing to GIS, but the use of breath testing to diagnose malabsorption may not be reliable [78].

### Nutrition Considerations with a Low FODMAP Diet

Depending on the extensiveness of high FODMAP food restriction, several complications associated with a strict long-term low FODMAP diet have been identified. A low FODMAP diet may be associated with alterations in gut microbiota, reduced short-chain fatty acid production, and impacts on aspects of physical and psychological wellbeing, similar to those mentioned in Sect. 2.5 with regard to a GFD [75, 76, 81]. Diminished concentrations of *bifidobacteria* after 3–4 weeks of reduced FODMAP intake have been observed [76, 81]. Exercise may neutralize the adverse effects of diminished prebiotic consumption on the microbiota. If adherence to FODMAP restriction is periodic/short-term, or limited to a few foods, altered microbiota is less concerning [82]. Coadministration of a multistrain probiotic has also been shown to mitigate the detrimental effects of lowered prebiotic intake [83, 84]. In addition, short-chain fatty acid production, which is highly reliant on fermentation of undigested carbohydrates in the large intestine [85, 86], may theoretically be compromised with reduced substrate. However, after 21 days of a low FODMAP diet, short-chain fatty acid fecal concentrations were similar between the low FODMAP and control diets [81]. A multitude of dietary and environmental factors modulate the composition and metabolic activity of the gut microbiota. Research on the microbiota in clinically healthy athletes implementing a low FODMAP diet has not been published; however, it is prudent to consider this aspect of gut health with frequent or long-term FODMAP restriction.

### Practical Applications of a Low FODMAP Diet for Athletes

**Fig. 2:**
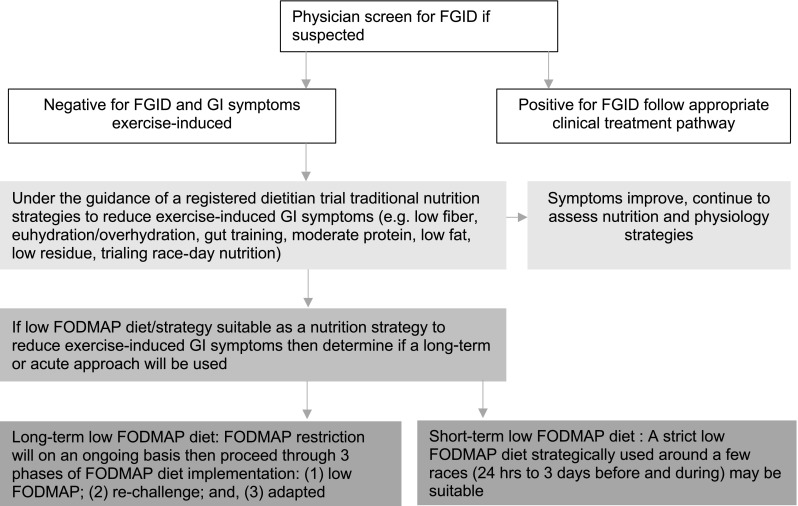
Proposed brief decision pathway for the use of a FODMAP restriction diet for the treatment of exercise-induced gastrointestinal symptoms as part of a nutritional management plan. *FGID* functional gastrointestinal disorder, *GI* gastrointestinal, *FODMAP* fermentable oligo-, di-, monosaccharides and polyols

For some athletes, supporting optimal nutrient intake and fueling for exercise while adhering to a low FODMAP diet may be challenging [75, 87]. Dietitian-led support from a professional specially trained in sports nutrition and the low FODMAP diet may facilitate proper use of the diet [88]. If a long-term low FODMAP diet is warranted, the three phases of the diet, as designed by the gastroenterology group at Monash University (Melbourne, VIC, Australia), should be followed [74, 88]. Transferring this clinical diet to a healthy athletic population will likely not necessitate the strictest form of the diet. For example, lactose or fructose may be the only symptom triggers [13]. Therefore, reduction of high lactose and excess fructose-containing foods (e.g. cow’s milk and some fruits or sports foods) that are habitually consumed may be the only modifications required for symptom improvement. Trigger foods may only elicit symptoms when ingested before or during strenuous exercise, such as racing. In this case, practical evidence suggests reduction of these foods will only be required 1–3 days before and during intensive endurance exercise. Using this concept, a 3 day low FODMAP diet has been implemented based on the idea that a minimal period of 24 h is required to eliminate short-chain carbohydrates from the GI tract [61, 62]. During the immediate post-exercise recovery phase, when optimal nutrient delivery is important but may be compromised, reduction of high FODMAP foods may also be warranted [11]. Figure 2 summarizes a notional decision-making process for sports nutrition practitioners integrating a low FODMAP diet into a treatment plan for exercise-induced gastrointestinal syndrome.

FODMAP diet application in the athletic arena is in its infancy; however, the development of low FODMAP sports food products/energy bars, as well as oral nutrition supplements [89], will support adherence to sports nutrition guidelines alongside FODMAP restriction. Introduction of a low FODMAP diet must be carefully navigated to minimize the risks associated with unnecessary dietary restriction (Fig. 2) [75, 90, 91]. It is imperative that practitioners consider underlying clinical conditions, such as functional gastrointestinal disorders (e.g. IBS, Crohn’s disease, functional idiopathic nausea), and the potential for unnecessary food restriction to foster eating disorders.

## Conclusions

Novel nutrition strategies continue to emerge that will enhance our understanding of the interplay between exercise-associated gastrointestinal dysfunction and diet. Although no supportive evidence exists, GFDs have gained far-reaching recognition as a successful strategy to reduce exercise-associated GIS and improve parameters influencing athletic performance. Emerging evidence suggests that the modulation of GIS reported with reduction of FODMAPs concomitant with elimination of gluten-containing grains may be secondary to reduction of FODMAPs rather than gluten. Furthermore, and of relevance to the clinical application of a low FODMAP diet to treat IBS symptoms, there is increasing awareness that malabsorption is a potential factor contributing to exercise-associated GIS. The etiology of GIS remains complex, but advancing work offers preliminary insight into the application of FODMAP manipulation for management of exercise-induced gastrointestinal syndrome. Evolving applied research and informed practitioners will be essential for the effective integration of low FODMAP strategies to address the multifactorial etiology of gastrointestinal issues in athletes.
